# Hard X-ray polarizer to enable simultaneous three-dimensional nanoscale imaging of magnetic structure and lattice strain

**DOI:** 10.1107/S1600577516009632

**Published:** 2016-08-04

**Authors:** Jonathan Logan, Ross Harder, Luxi Li, Daniel Haskel, Pice Chen, Robert Winarski, Peter Fuesz, Deborah Schlagel, David Vine, Christa Benson, Ian McNulty

**Affiliations:** a Center for Nanoscale Materials, Argonne National Laboratory, 9700 South Cass Avenue, Argonne, IL 60439, USA; b Advanced Photon Source, Argonne National Laboratory, 9700 South Cass Avenue, Argonne, IL 60439, USA; c Department of Materials Science and Engineering, Northwestern University, 2220 Campus Drive, Evanston, IL 60208, USA; d Division of Materials Science and Engineering, Ames Laboratory, 2405 Kooser Drive, Ames, IA 50011, USA; e Lawrence Berkeley National Laboratory, Berkeley, CA 94720, USA

**Keywords:** Bragg coherent diffractive imaging, XMCD, nanomagnetism, strain

## Abstract

Recent progress in the development of dichroic Bragg coherent diffractive imaging, a new technique for simultaneous three-dimensional imaging of strain and magnetization at the nanoscale, is reported. This progress includes the installation of a diamond X-ray phase retarder at beamline 34-ID-C of the Advanced Photon Source. The performance of the phase retarder for tuning X-ray polarization is demonstrated with temperature-dependent X-ray magnetic circular dichroism measurements on a gadolinium foil in transmission and on a Gd_5_Si_2_Ge_2_ crystal in diffraction geometry with a partially coherent, focused X-ray beam. Feasibility tests for dichroic Bragg coherent diffractive imaging are presented. These tests include (1) using conventional Bragg coherent diffractive imaging to determine whether the phase retarder introduces aberrations using a nonmagnetic gold nanocrystal as a control sample, and (2) collecting coherent diffraction patterns of a magnetic Gd_5_Si_2_Ge_2_ nanocrystal with left- and right-circularly polarized X-rays. Future applications of dichroic Bragg coherent diffractive imaging for the correlation of strain and lattice defects with magnetic ordering and inhomogeneities are considered.

## Abstract 2

The performance of a diamond X-ray phase retarder to enable the production of circularly polarized X-rays has been quantitatively measured and magnetic dichroism contrast in transmission and diffraction geometries has been demonstrated. Feasibility tests for dichroic Bragg coherent diffractive imaging experiments were performed and showed that the diamond X-ray phase retarder does not produce significant distortions to the X-ray wavefront and that Bragg coherent diffractive imaging reconstructions are achievable.

## 1. Introduction   

Understanding how magnetic ordering couples to electronic, structural and orbital degrees of freedom lies at the frontier of condensed matter physics. Key questions to investigate are how spins couple to the underlying atomic lattice order and how this coupling evolves at phase changes, such as magneto-structural phase transitions. Often, elastic strain mediates the interaction between the magnetic and other degrees of freedom. During phase transitions, different magnetic phases can nucleate and grow at defect sites due to the effects of local lattice strains on the free-energy landscape (Evans *et al.*, 2002 ▸). Similarly, local strain fields have a significant impact on equilibrium magnetic domain configurations (Moore *et al.*, 2009 ▸; Wu *et al.*, 2001 ▸). However, little is quantitatively known about the correlation between lattice strain texture and spin texture at domain boundaries at the nanoscale.

There is also a growing technological interest in the effects of lattice structure and strain on the magnetic properties of nanometer-scale devices. Recently, there has been a push towards voltage-controlled magnetic devices consisting of a ferromagnetic layer coupled to an electrosensitive layer (Morosov, 2014 ▸). However, in these composite multiferroic devices it is difficult to unravel whether electric field control over ferromagnetism arises from elastic strain transfer or from other phenomena such as interfacial exchange bias. It would therefore be invaluable to collect magnetic information simultaneously (*e.g.* element-specific magnetization loops, preferred magnetization directions, orbital and spin moments) with structural information (*e.g.* crystal structure, defects, dislocations, lattice strain) from individual sub-micrometer-sized objects.

Synchrotron-based hard X-ray sources have enabled revolutionary breakthroughs for probing magnetism and strain at the nanoscale. Ferromagnetism is customarily probed with circularly polarized X-rays using resonant techniques (Lovesey & Collins, 1996 ▸; Stöhr & Siegmann, 2006 ▸). The use of circularly polarized X-rays enables interference between electron-density scattering and magnetic scattering. Techniques for studying nanoscale ferromagnetism have utilized this charge–magnetic interference phenomenon with polarized X-rays to obtain magnetic sensitivity. In the absence of interference, the resonantly scattered magnetic intensity is typically 10000 times weaker than the charge scattering intensity. Using linearly polarized X-rays one would need very precise polarimetry to differentiate between charge and magnetic signals in the diffracted intensity. In contrast, using circularly polarized X-rays, the charge–magnetic interference effect can be several percent of the total intensity. Therefore, it has been utilized to attain sub-monolayer magnetic sensitivity in thin films as well as sensitivity to sub-unit-cell and ensemble-averaged magnetic features of crystals (*e.g.* site-specific measurements of inequivalent Nd sites in Nd_2_Fe_14_B crystals) (Haskel *et al.*, 2005 ▸, 2012 ▸). Nanoscale strain imaging has also evolved rapidly in the past decade, since the adaptation of X-ray coherent diffractive imaging (CDI) techniques to the Bragg diffraction geometry. Bragg coherent diffractive imaging (BCDI) enables the investigation of three-dimensional morphology, electron density and strain inside compact, crystalline objects (Robinson & Harder, 2009 ▸; Clark *et al.*, 2012 ▸) at length scales to the 10 nm scale, which is below the resolution of X-ray lenses. By using X-ray magnetic circular dichroism (XMCD) in the diffraction geometry, we gain the ability to study magnetism and perform BCDI strain mapping with a single X-ray probe.

Circularly polarized X-rays can be directly generated using a helical undulator, accessed from above or below the orbital plane of a bending magnet radiation, or produced from linear­ly polarized X-rays using phase-retarding optics. X-ray phase retarders (XPRs) offer several important advantages for generation of circularly polarized X-rays in the hard X-ray (>3 keV) regime. They achieve a high degree of circular polarization, are tunable over a wide energy range and allow fast polarization switching (Suzuki *et al.*, 2014 ▸). XPRs are commonly made from diamond due to low attenuation of hard X-rays and the availability of nearly perfect single crystals. Thus, diamond XPRs have been widely used at synchrotron light sources (Mills, 1997 ▸; Giles *et al.*, 1994 ▸; Lang & Srajer, 1995 ▸; Suzuki *et al.*, 2014 ▸). On the other hand, it is not obvious that coherent X-ray methods depending on use of a well defined, stable wavefront are possible using XPR optics (Hruszkewycz *et al.*, 2010 ▸).

Here, we describe a diamond XPR setup at the 34-ID-C beamline of the Advanced Photon Source and quantify its performance. Our motivation for installing the XPR at 34-ID-C is to image magnetic structure in individual nanocrystals in addition to crystal structure and lattice strain by BCDI. We demonstrate that this XPR setup can accurately tune polarization of incident X-rays for magnetic dichroism measurements. Examples of temperature-dependent XMCD spectra are illustrated in transmission as well as diffraction geometry. Lastly, we describe feasibility tests for dichroic BCDI, a technique for simultaneous measurements of three-dimensional strain and magnetization in nanocrystals. We show that the X-ray wavefront passing through the XPR is sufficiently coherent and has long-time stability for BCDI phase reconstruction. Additionally, we demonstrate that preliminary coherent diffraction patterns on a 200 nm-scale magnetic crystal show small intensity differences when collected with left- or right-circularly polarized X-rays. These differences are likely due to a combination of the helicity dependent charge–magnetic interference scattering and experimental noise.

## 2. Polarizer theory, design and performance   

When the diamond crystal in an XPR is tuned near a Bragg condition, the σ and π polarization components of the incident beam electric vector propagate with different phase velocities inside the crystal. This results in a phase difference δ that depends on the angular deviation of the crystal from the ideal Bragg condition, Δθ = θ − θ_B_, as well as the thickness *t* of the phase retarding crystal, according to (Suzuki *et al.*, 2014 ▸)
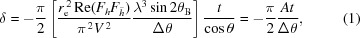
where *r*_e_ is the classical electron radius, 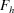 and 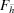 are the structure factor for the *hkl* and 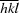 reflections, respectively, *V* is the unit cell volume, λ is the X-ray wavelength, θ_B_ is the Bragg angle and *A* = 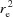
Re(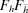)λ^3^(sin 2θ_B_/π^2^ *V* ^2^cos θ). The phase shift is related to the degree of linear and circular polarization in the transmitted beam by
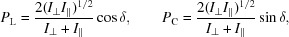
where 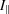 and 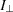 are the intensities of the polarization components in and out of the diffraction plane. To achieve a circularly polarized beam, these intensities must be the same, and δ must be equal to π/2. Equal intensities can be obtained by inclining the diffraction plane of the phase retarder at 45° with respect to linear polarization of the incoming beam. Because the phase difference is a function of Δθ, the polarization of the transmitted beam is easily adjusted by tuning Δθ to achieve the desired phase shift δ. Therefore, rocking around a diamond Bragg peak enables conversion of horizontal linearly polarized X-rays into left-circularly (LCP) or right-circularly (RCP) polarized X-rays.

**Figure 1:**
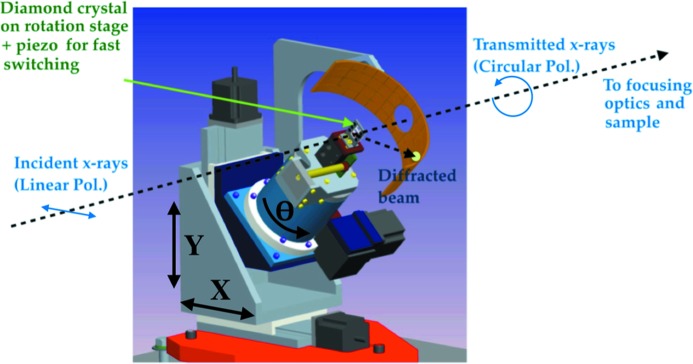
Illustration of the diamond X-ray phase retarder installed at APS beamline 34-ID-C at Argonne National Laboratory.

We installed a diamond XPR instrument (shown in Fig. 1 ▸) in the 34-ID-B station following the Si(111) double-crystal monochromator for the 34-ID-C beamline. The XPR uses the Bragg transmission geometry that enables switching polarization states without deviating the X-ray beam (Lang & Srajer, 1995 ▸; Winarski *et al.*, 2012 ▸).

The XPR accommodates up to three diamond crystals in the beamline vacuum. Crystals are inserted into the incident X-ray beam and aligned to the (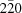) Bragg reflection of the diamond crystals on a three-axis (*XY*θ) stage. A servo-controlled piezoelectric linear translator is used for fine adjustment of the diamond rotation angle with an angular resolution of ∼30 µrad. This piezo-driven fine-rotation axis enables up to kilohertz switching between LCP and RCP modes. A scintillation screen and video camera built into the XPR instrument enable rapid location of the Bragg peak over a wide range of X-ray energies. We mounted three (111)-oriented diamonds in the XPR with thicknesses of 400 µm (Sumitomo), 200 µm and 100 µm (Delaware Diamond Knives). The optimum choice of diamond thickness depends on the X-ray energy according to equation (1); thicker diamonds perform better at higher X-ray energies (Suzuki *et al.*, 2014 ▸).

**Figure 2:**
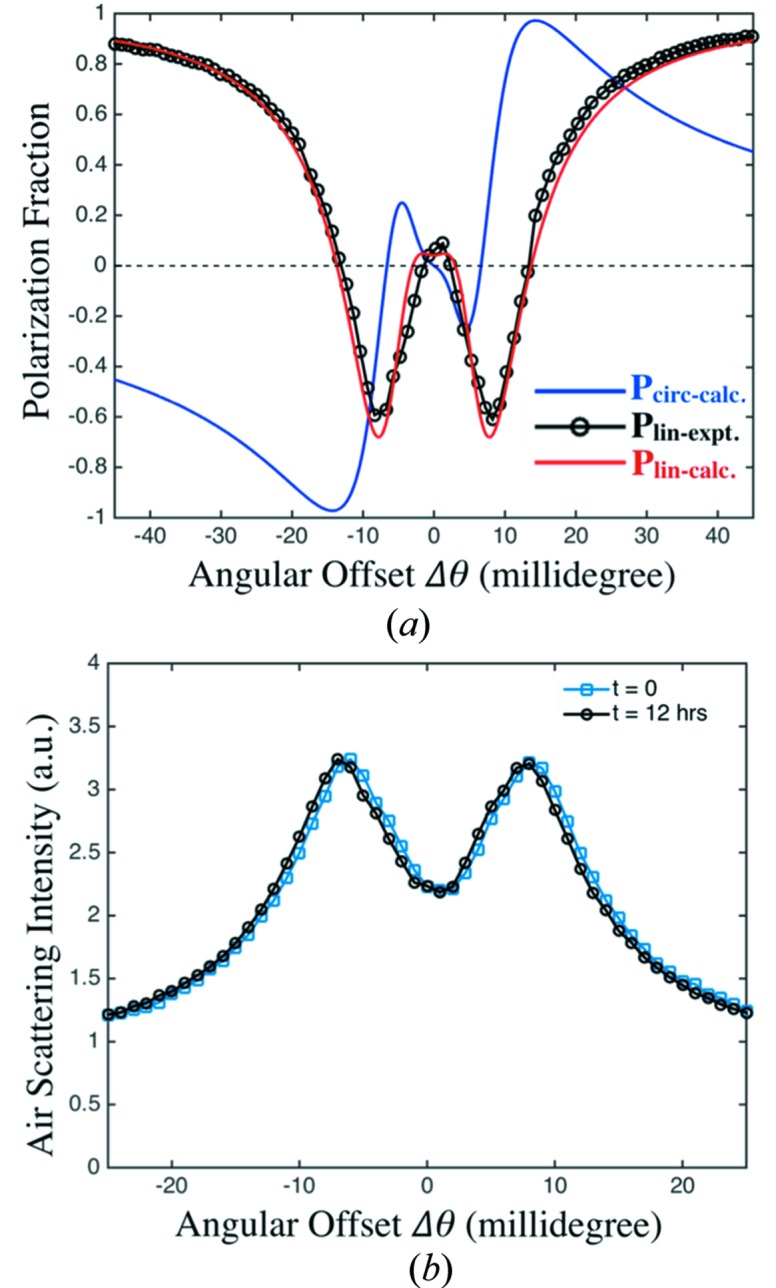
(*a*) Linear and circular polarization fraction as a function of X-ray phase retarder angular offset, where Δθ is the deviation from the ideal Bragg condition. The linear (red curve) and circular (blue curve) polarization fraction are calculated for a monochromator bandwidth of Δ*E*/*E* = 1.07 × 10^−4^. Black circles show experimental values for the linear polarization measured with a Ge(333) analyzer crystal at 8 keV. (*b*) Air-scattered intensity measured before and after a 12 h time interval. Changes correspond to a slow drift in the XPR alignment of 0.001° in 12 h.

Polarimetry data were collected at 8 keV using a Ge(333) analyzer crystal located downstream of the diamond polarizer. This setup, described in detail by Lang & Srajer (1995 ▸), allowed us to measure the linear Stokes–Poincaré parameters directly. Fig. 2 ▸(*a*) shows the measured results for the linear polarization, as well as theoretical calculations for linear and circular polarization assuming a monochromator bandwidth of Δ*E*/*E* = 1.07 × 10^−4^ (Leake *et al.*, 2009 ▸).

During typical operation we obtained real-time feedback on the polarization state incident on the samples by monitoring X-rays elastically scattered from the air using Oxford NaI scintillation detectors oriented to the horizontal and vertical axes. The linear polarimetry data shown in Fig. 2 ▸(*b*) indicate a high level of polarizer stability with a drift of only one millidegree over 12 h. We periodically compensated for this slow drift by tweaking the angle of the diamond crystal based on the polarimetry data.

## 3. Magnetic dichroism results   

**Figure 3:**
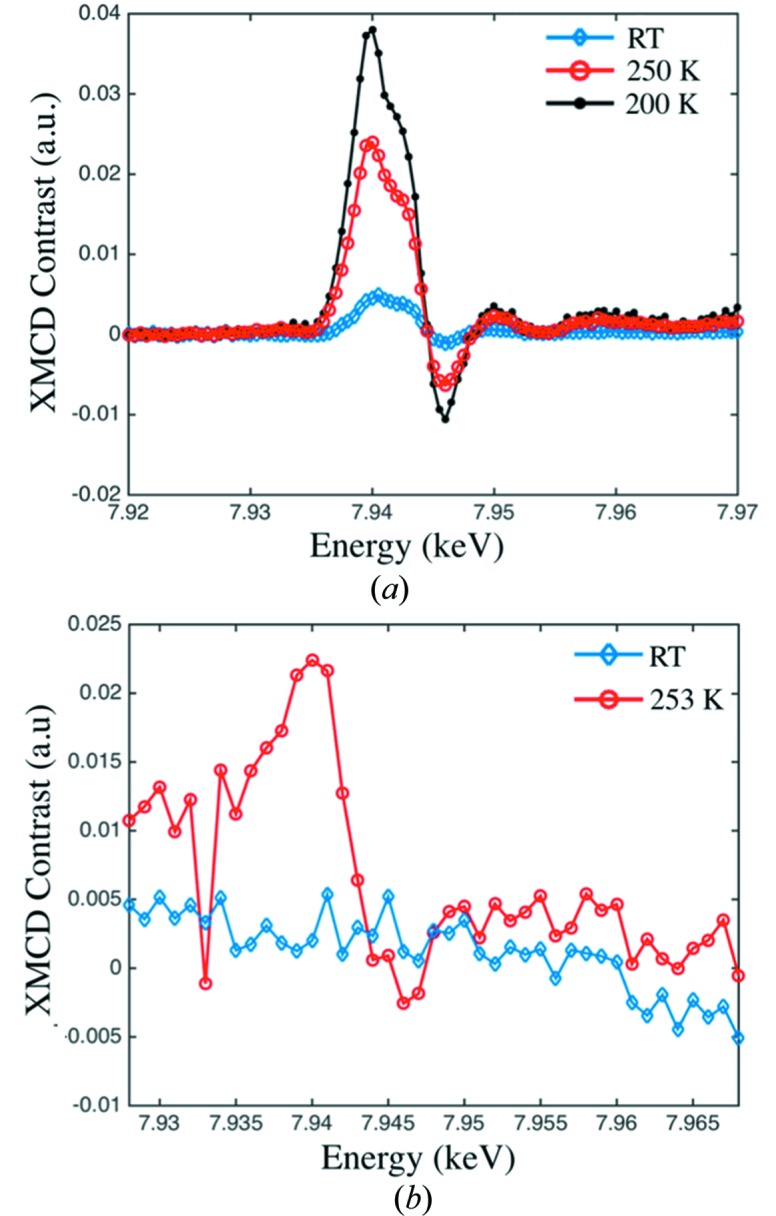
(*a*) Transmission XMCD measured through a 5 µm-thick Gd foil at 200 K (black dots), 250 K (red circles) and room temperature (blue diamonds). (*b*) Diffraction XMCD measured from a Gd_5_Si_2_Ge_2_ *b*-axis-oriented single-crystal using a focused coherent X-ray beam at 253 K (red circles) and room temperature (blue diamonds).

We demonstrated the performance of the XPR with magnetic contrast measurements in both the transmission and the diffraction geometries. X-ray magnetic circular dichroism (XMCD) spectra were acquired with a PIN photodiode as a function of energy across the Gd *L*_2_-absorption edge through a 5 µm-thick Gd foil in the transmission geometry. Bulk Gd orders as a ferromagnet below a Curie temperature of 293 K. The foil was magnetized in-plane by placing it in the 0.3 T field of a permanent magnet assembly and cooled to various temperatures using an Oxford Cryostream 700. We oriented the foil at approximately 45° to the incident beam so that the magnetic absorption contrast, which is proportional to 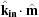, remains sizable while the Gd effective thickness only increases to 5 × 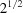 = 7.07 µm. Fig. 3 ▸(*a*) shows the XMCD contrast for several temperatures obtained with helicity switching during each point of an energy spectrum with an unfocused X-ray beam.

Next, we used a Medipix 2 detector to measure the dichroism signal from a *b*-axis-oriented Gd_5_Si_2_Ge_2_ single-crystal using a partially coherent X-ray beam focused to 0.6 µm FWHM by a Kirkpatrick–Baez mirror pair. The Gd_5_(Si_*x*_Ge_1−*x*_)_4_ system is a well known family of giant-magnetocaloric materials that show promise for room-temperature magnetic refrigeration (Pecharsky & Gschneidner, 1997 ▸). The Gd_5_Si_2_Ge_2_ composition has a Curie temperature of 276 K. We measured XMCD spectra across the Gd *L*_2_-absorption edge at the (040) Bragg diffraction peak. The XMCD contrast, (LCP − RCP)/(LCP + RCP), measured at room temperature and 253 K, on either side of the main magneto-structural phase transition at ∼277 K, is displayed in Fig. 3 ▸(*b*).

## 4. Dichroic Bragg coherent diffractive imaging: feasibility tests   

Dichroic BCDI is a technique we are developing to enable three-dimensional strain and magnetization profiling with a single X-ray probe. Using dichroic BCDI, we collect BCDI datasets with both LCP and RCP X-rays and separately reconstruct the nanocrystal sample in the two cases. By taking the difference between the reconstructed LCP and RCP magnitudes, the ‘pure’ electron density term can be removed to isolate the magnetic component of the object structure. We performed two measurements to test the feasibility of dichroic BCDI experiments.

**Figure 4:**
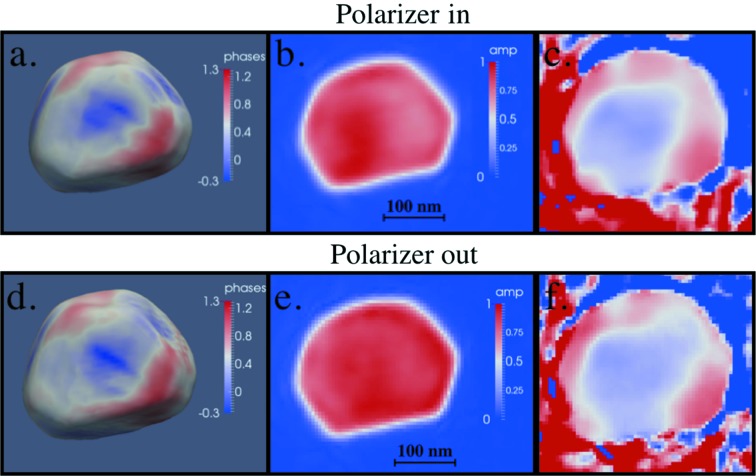
Top row: gold nanocrystal reconstruction with diamond polarizer in the beam showing (*a*) the magnitude of the complex amplitude as a 25% threshold isosurface where hue indicates the phase of the complex amplitude in radians, (*b*) reconstructed magnitude of a slice through the nanocrystal near the crystal center, (*c*) reconstructed phase of the same slice through the nanocrystal as seen for the magnitude slice in (*b*). Bottom row: reconstruction of the same gold nanocrystal with XPR taken out of the beam, showing (*d*) the magnitude of the complex amplitude as a 25% threshold isosurface where hue indicates the phase of the complex amplitude in radians, (*e*) reconstructed magnitude of a slice through the nanocrystal near the crystal center and (*f*) reconstructed phase of the same slice through the nanocrystal as seen for the magnitude slice in (*e*).

For the first feasibility measurement, we tested whether the X-ray wavefront passing through the XPR optics was stable and sufficiently defined for BCDI experiments by recording BCDI datasets from a nonmagnetic ∼200 nm Au nanocrystal with and without the XPR in the X-ray beam. Reconstructions of the BCDI data obtained at the Au (111) Bragg peak indicate only minor differences (Fig. 4 ▸). These small differences may be partially due to X-ray wavefront aberrations caused by the XPR or, more likely, the limited ability of the iterative algorithm used to recover the missing object phase to fully converge. Nevertheless, this test illustrates that the diamond crystal we used is of high enough quality to sufficiently preserve the X-ray wavefront to allow BCDI reconstructions with magnetic sensitivity and obtain accurate information on the amplitude and strain of individual nanocrystals.

**Figure 5:**
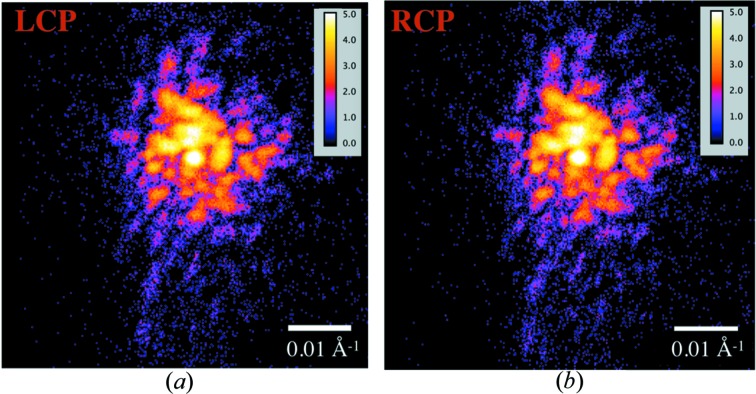
The coherent diffraction pattern near the center of the (301) Bragg peak rocking curve from a Gd_5_Si_2_Ge_2_ nanocrystal at 253 K using (*a*) LCP and (*b*) RCP X-rays, shown with a logarithmic color scale. Because the helicity dependent magnetic contrast from ‘C–M’ interference is less than 2% of the ‘charge-only’ signal, the two diffraction patterns look almost identical to the eye.

For the second feasibility measurement, we recorded coherent diffraction patterns at the (301) Bragg peak from individual ∼200 nm Gd_5_Si_2_Ge_2_ crystals at 253 K. The crystals, obtained from a Gd_5_Si_2_Ge_2_ powder sample, were coated with 500 nm of Cr to secure them to a Si substrate. The diffraction measurements were performed near the Gd *L*_2_-absorption edge where the maximum XMCD signal was observed in Fig. 3 ▸(*b*). Diffraction patterns of a ∼200 nm Gd_5_Si_2_Ge_2_ crystal, taken with LCP as well as RCP X-rays, are displayed on a logarithmic color scale in Fig. 5 ▸. These diffraction patterns consist of ‘charge-only’ signal as well as the ‘charge–magnetic (C–M) interference’ signal that provides magnetic contrast. LCP and RCP X-rays diffracting from a ferromagnetic crystal have the same ‘charge-only’ intensity, but the ‘C–M interference’ intensity components have opposite sign. Since the ‘C–M interference’ term is only 1–2% of the ‘charge-only’ term for Gd_5_Si_2_Ge_2_ at this temperature, we would expect that the LCP and RCP intensities would look virtually equivalent. This expectation is confirmed in Fig. 5 ▸. The normalized cross-correlation coefficient for the LCP and RCP patterns was 0.9764, indicating a high degree of correlation between them. The minor differences result from a combination of the C–M interference signal of interest, and sources of noise (*e.g.* counting statistics, or slow drifts of sample, beam and polarizer). We found that the coherent diffraction data in Fig. 5 ▸ were too complicated to be inverted with the phase retrieval algorithm we used (Clark *et al.*, 2012 ▸). BCDI phase retrieval techniques work best when there are relatively few defects that cause deviations from the ideal crystalline order. In future measurements we plan to use faceted magnetic nanocrystals of CoPt, SmCo_5_, Gd or GdFe_2_ for more facile reconstructions.

## 5. Conclusions   

We installed a diamond X-ray phase retarder at beamline 34-ID-C of the Advanced Photon Source and quantified its performance with polarimetry measurements. We then demonstrated its operation with magnetic dichroism measurements. This included measuring the temperature-dependent XMCD signal from Gd foil in transmission geometry and the XMCD signal from a Gd_5_Si_2_Ge_2_ crystal using a partially coherent, focused beam in diffraction geometry. Finally, we performed two feasibility tests for future dichroic BCDI experiments. The first of these tests showed that the X-ray wavefront is sufficiently preserved upon transit through the diamond phase retarder crystal to allow BCDI reconstructions. In the second test, we collected coherent diffraction patterns from a single ∼200 nm Gd_5_Si_2_Ge_2_ nanocrystal with left- and right-circularly polarized X-rays at 253 K. The coherent diffraction patterns obtained with LCP and RCP X-rays appear virtually identical, apart from slight differences that may arise from the expected charge–magnetic interference contrast.

We expect to have access to a wide range of magnetic and structural information in nanosystems, with future integration of a variable magnetic field apparatus into the experiment. Realisation of dichroic BCDI with a focused, coherent X-ray beam will enable us to obtain magnetic information such as element-specific hysteresis loops and preferred magnetization directions from a single nanocrystal and explore how its magnetic properties vary as a function of shape, size, defect structure and strain. It will also enable us to examine nano­scale phenomena such as magnetic return-point memory to determine the effects of the local defects on the pinning landscape. These experiments will benefit dramatically from the planned hundredfold brightness increase envisioned by the APS-U upgrade. We anticipate this work will pave the way forward for experiments to utilize BCDI in unique and powerful ways.
